# Relationship between Degree of Deformation in Quartz and Silica Dissolution for the Development of Alkali-Silica Reaction in Concrete

**DOI:** 10.3390/ma10091022

**Published:** 2017-09-04

**Authors:** Francieli Tiecher, Marcia E. B. Gomes, Denise C. C. Dal Molin, Nicole P. Hasparyk, Paulo J. M. Monteiro

**Affiliations:** 1 Polytechnic School of Civil Engineering, IMED, Passo Fundo 99070-220, Brazil; 2 Department of Geology, Universidade Federal do Rio Grande do Sul, Porto Alegre 91501-970, Brazil; marcia.boscato@ufrgs.br; 3 Department of Civil Engineering, Universidade Federal do Rio Grande do Sul, Porto Alegre 90010-281, Brazil; dmolin@ufrgs.br; 4 Department of Services and Technological Innovation of FURNAS Centrais Elétricas S. A., Goiânia 74984-670, Brazil; nicolepha@gmail.com; 5 Department of Civil and Environmental Engineering, University of California, Berkeley, CA 94720, USA; monteiro@berkeley.edu

**Keywords:** alkali-silica reaction, quartz, silica dissolution, deformation

## Abstract

This paper presents research on the influence of quartz deformation in aggregates for the development of the alkali-silica reaction in concrete and its relationship with silica dissolution. The study also compares these characteristics with the field behavior of such rocks in concrete. The paper proposes parameters to classify the different degrees of deformation of quartz. Transmission electron microscopy showed the presence of walls even in slightly deformed quartz, which indicate the presence of the internal paths available to react with the alkaline concrete pore solutions and point to the potential development of an alkali-silica reaction. The presence of the deformation bands in the quartz grains leads to the alkali aggregate reaction occurring more rapidly. The visible spectrophotometer test was performed to evaluate the dissolution potential of the different samples of deformed quartz, which confirmed that the reactivity of the quartz increases as the deformation of the crystalline structure increases. The parameters established in the present study could be verified by analyzing the behavior of reactive and innocuous aggregates from the buildings.

## 1. Introduction

The chemical reaction which occurs between the silica found in aggregates and the alkali hydroxides released during hydration of cement is called an alkali-silica reaction (ASR). This reaction leads to the formation of an alkali-silica hygroscopic gel that expands when water is present. The increase in volume causes tensile stresses, which leads to the concrete cracking.

Quartz is the most abundant form of silica in concrete aggregates and for this reason quartz can be considered mainly responsible for ASR. The propensity of quartz to react is related to the features of its crystal structure, since under tectonic tensions it ‘displaces’ and ‘deflects’. Passchier and Trouw [1] explain that crystals have a certain internal energy, which is very small when it is free of displacements (perfect crystal-flawless). According to Wenk et al. [2], when a crystal of quartz is deformed there is an increase in displacements and in internal energy, which alters the distance between atoms. This energy boost is proportional to the increase in the length of the displacements by volume of crystalline material, and is called displacement density.

The imperfections in the crystal structure can be identified by undulatory extinction, which is a feature strongly related to the potential reactivity of minerals, especially quartz. There are criticisms regarding the use of this characteristic, since it is a subjective parameter and very difficult to measure using thin sections with common petrographic microscopes, especially when analyzing very deformed grains. However, several authors consider this to be one of the main aspects to be observed when classifying the potential reactivity of quartz [3,4,5,6,7,8,9,10,11].

On the other hand, many studies have shown that microcrystalline quartz grains are more reactive and promote greater silica dissolution than grains with intense undulatory extinction [12,13,14]. However, the continuous increase in the displacements of the crystal leads to a clustering of the networks, known as sub-grain walls which, with the boost in tensions, can break the original grain and thus form new recovered grains, that is, grains which do not have displacements.

In order to understand these differences, sometimes observed in the literature, this paper sets out to show some relations between the potential for ASR occurring and the mineralogical characteristics of quartz with different degrees of deformation. Mineralogical parameters to quantify the different degrees of deformation of the quartz grains are suggested. These parameters will be used to establish correlations between different degrees of quartz deformation and the susceptibility of rocks to developing ASR. Different methodologies will be used, among which is the visible spectrophotometer test, where the dissolution rate of the silica in alkaline solution is evaluated, based on the degree of deformation of the quartz. This methodology has not yet been recorded in the literature for evaluating the dissolution of silica in reactive aggregates.

The results obtained in this study were compared with the behavior of rocks in the field and this enabled it to be verified that quartz grains featuring deformation bands are more soluble. 

## 2. Materials and Methods

### 2.1. Materials

This study contemplates the evaluation of the characteristics of the quartz for the occurrence of ASR, so a quartzite rock containing 85% of quartz was selected [15]. This rock was used as coarse aggregate in concrete of the Furnas Hydroelectric Power Plant (Brazil), which was affected by ASR in 1976, 13 years after the plant was built [15]. A mylonite from the Metropolitan Area of Recife was also assessed. This aggregate exhibited deleterious processes caused by ASR in building foundations [16]. Moreover, a rock with deformed quartz grains previously categorized with an innocuous field behavior was used as a reference material (granite, from Metropolitan Area of Recife) [17].

Also, three rocks from the Monte Bonito (MB) outcrop [18] were selected. These rocks contain quartz grains with different degrees of deformation: MB granite, MB protomylonite and MB orthomylonite. They were collected from the same outcrop in order to compare only the reactivity/texture relation of the quartz grains containing different intensities of the deformation, without considering the influence of other characteristics, such as its chemical composition. Visual inspection was used for the selection.

In the Section 2.2.1 (Petrography analyses) was described the methodology to obtain the petrographic characteristics of the rocks used in this study. The petrographic characteristics of the MB granite, MB protomylonite and MB orthomylonite are shown in Table 1. Table 2 shows the petrographic characteristics of the reactive quartzite, reactive mylonite and innocuous granite.

**Figure 1:**
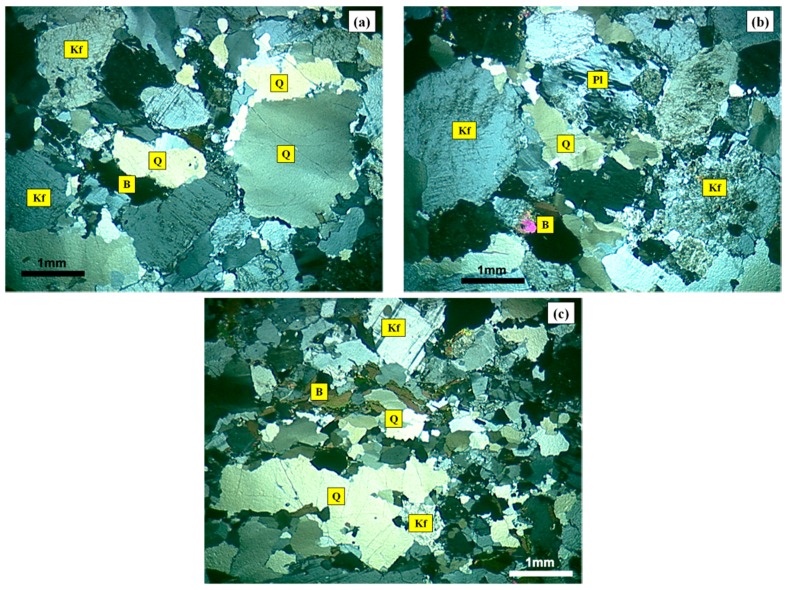
Photomicrographs of thin section under cross-polarized transmitted light microscopy of the Monte Bonito (MB) granite (**a**), MB protomylonite (**b**) and MB orthomylonite (**c**): Q = quartz; Kf = K-feldspar; B = biotite; Pl = plagioclase.

**Figure 2:**
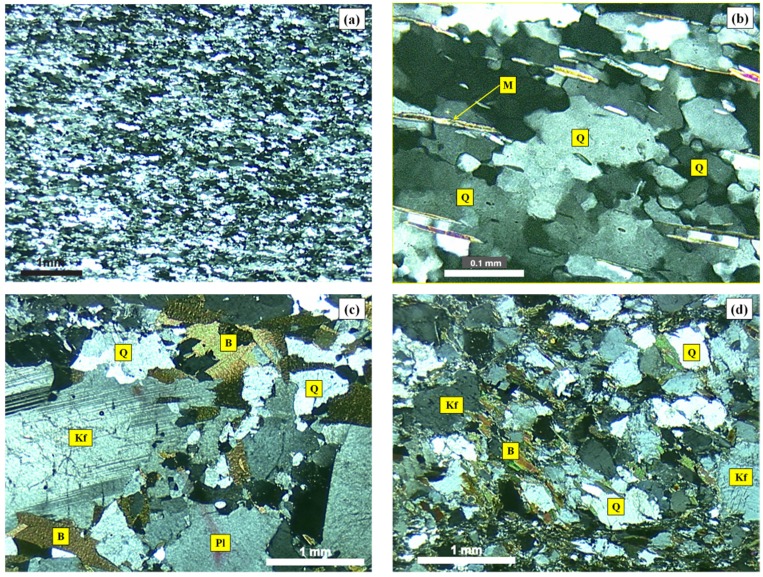
Photomicrographs of thin section under cross-polarized transmitted light microscopy of the quartzite (**a**,**b**), innocuous granite (**c**) and reactive mylonite (**d**): Q = quartz; Kf = K-feldspar; Pl = plagioclase; B = biotite; Pl = plagioclase; M = mica.

Figure 1 shows the micrographic images obtained by cross-polarized transmitted light microscopy of the MB granite, MB protomylonite and MB orthomylonite. Figure 2 shows the images of the reactive quartzite, reactive mylonite and innocuous granite.

### 2.2. Methods

#### 2.2.1. Petrography Analyses

Petrography analyses together with quantitative modal analyses were performed in order to characterize and quantify the constituent minerals of the rocks, with an emphasis on the texture of the mineral quartz. Thin sections (30 µm) were placed on an optical transmitted light microscope and a score of 2000 points per sample was obtained. The counts were repeated 5 times. Samples of mortar bars subjected to the accelerated mortar bar test were also observed by using petrography for the investigation. The accelerated mortar bar test was described in the Section 2.2.3.

#### 2.2.2. Transmission Electron Microscopy

Transmission electron microscopy (TEM, Jeol–JEM 2010, Peabody, MA, USA) was used to characterize the degree of deformation of quartz grains. The analyses were performed similarly to the investigations carried out by Wenk et al. [2], but they did not measure the displacement density of the grains.

The samples were selected to be representative of the different degrees of deformation of quartz grains as revealed by petrographic analysis. The selected grains were analyzed using a Jeol–JEM 2010 microscope. Thin 30 μm-thick slides were made with a double face, and fixed with adhesive soluble in acetone. From these slides, grains representative of the different degrees of deformation were selected using petrographic analysis. These grains were extracted from the matrix, the region marked on the slide being cut out with an ultrasound apparatus and then submerged in acetone. Thus, samples of approximately 3 mm in diameter and 30 μm thick of the selected grains were obtained. Then electro-polishing in the ion-mill was carried out until the samples were sufficiently thin for the transmission electrons to be visualized, that is, they were approximately 1μm thick.

#### 2.2.3. Accelerated Mortar Bar Method

The expansion test, the accelerated mortar bar test that was specified in ASTM C 1260 [19], was used to quantify the alkali-reactivity of the samples. The results of this test and the mineralogical features of rocks indicate the most important characteristics to be considered when analyzing the potential reactivity of an aggregate.

For testing, rock samples were crushed to meet the sand grading requirements prescribed by the standard. Mortar bars with a width of 25 mm and a length of 285 mm were cast using an aggregate/cement ratio of 1:2.25 (cement:sand) and a water/cement ratio of 0.47. The cement used for this study was a Portland cement, with no admixture (alkali content (Na_2_O_eq_): 0.89%; fineness: 4.92 cm²/g; autoclave expansion: 0.02%).

Three test specimens were cast for each sample. The bars were kept in their molds for 24 h in the curing room at 100% RH. Then, they were removed from the molds and immersed in a water bath at 80 °C for another 24 h. After this storage period, the bars were removed from the water bath and an initial reading was taken. The bars were then fully submerged in 1 M NaOH solution at 80 °C. Length measurements were taken during the 30-day storage period.

Based on the average of the expansions of the three test specimens molded with the aggregates to be analyzed and the standard cement, the aggregates were classified as potentially reactive, when they expanded by more than 0.10% at 30 days [20], or as potentially innocuous when they expanded by a lesser amount.

#### 2.2.4. Chemical Method of ASTM C 289/2007

The chemical method was performed according to ASTM C 289/2007 [21]. The test used rock fractions finer than #0.30 mm and coarser than #0.15 mm, and was performed in triplicate with 25 g samples. Samples were stored in a stainless steel container with 25 mL of 1 M NaOH solution at 80 °C (one stainless steel container was used as a blank test). The method prescribed by ASTM C 289/2007 [21] requires an analysis to be made of the amount of dissolved silica and of the reduction in alkalinity in the alkaline solution after the aggregates have been exposed to it for 24 h. However, due to criticism by several authors of the reliability of this method [9,17,22], since many aggregates known to be reactive produce non-reliable results, in addition to the analysis at 24 h, longer periods of exposure to the alkaline solution were proposed, namely 72 h and 168 h.

At the end of these periods, the samples were filtered. Part of the resulting solution was used to determine the mass of the dissolved silica, and another part to assess the reduction in alkalinity (pH was evaluated by using titulometry).

#### 2.2.5. Visible Spectrophotometer Method

In order to determine the potential of dissolving silica from quartz, due to its degree of deformation, the visible spectrophotometer method was performed, following the Brazilian standard NBR 9848 [23], which is often used for the chemical analysis of water. This standard specifies the test method for determining silica (SiO_2_) in liquid caustic soda by means of a visible spectrophotometer using ammonium molybdate. The method determines the color changes resulting from a silicomolybdic complex, with a pH close to 1, formed through visible spectrophotometry, and a silica concentration proportional to the existing silica. A 680 nm wavelength was used in the equipment.

For the test, quartz grains were extracted from the rock matrices. The aim of this analysis was to verify the influence of the quartz deformation on the ASR, excluding the interference of other silicates that are known to contribute both to the dissolution of silica and with alkalis in the ASR. Mineral separation was done for rock samples with a grain size below #0.15 mm. Firstly, the micas were separated magnetically by means of a Frantz Isodynamic Magnetic Separator. Then, the separation between quartz and feldspars (K-feldspar and plagioclase) was performed by taking advantage of the difference in density between them. Thus, the quartz grains extracted from the rocks were immersed in 1 M NaOH solution at 80 °C for 3 days. After this period, the samples were filtered and the resulting solution was used in the analysis by the aforesaid method.

## 3. Results

### 3.1. Assessment of the Degrees of Deformation

In order to correlate the textural characteristics of the quartz grains occurring in the rocks that were prone to developing ASR, the methodology presented in Table 1 and Table 2 was used. Dollar-Mantuani [24] proposed a correlation of the angle of undulatory extinction of the quartz with its potential reactivity. According to the author, grains with an undulatory extinction angle greater than 25° are potentially reactive and when this is less than 15°, this denotes innocuous quartz. However, Dollar-Mantuani [23] emphasizes that this measure is not completely accurate in conventional optical petrography; thus, when the measure of undulatory extinction angle is used to define the reactivity of the aggregate, an uncertainty of up to 60% may occur.

Based on petrographic analyses, a methodology was applied to classify the different degrees of deformation of quartz that had occurred in the aggregates as a result of the development of ASR, as described in Table 3. The quartz grains with different degrees of deformation were quantified by quantitative modal analyses, as described on Section 2.2.1. Table 4 presents the results obtained for each rock. Note that the degree-2 of deformation is predominant in almost all samples, except in granites (MB granite and innocuous granite). Grains with degree-0 of deformation were only found in the innocuous granite tested.

As shown in Table 4, the amount of grains presenting intense undulatory extinction, forming sub-grains (degree-3 of deformation), is higher in the reactive quartzite compared to the other samples. The MB orthomylonite and the innocuous granite have a great amount of completely recrystallized grains.

The surface available for the reaction with the alkaline hydroxides grows when the quartz grains deform, since chemical links are weaker in the deformed zones. This weakening of the chemical bonds between the atoms leads to the grain subdividing [25].

**Figure 3:**
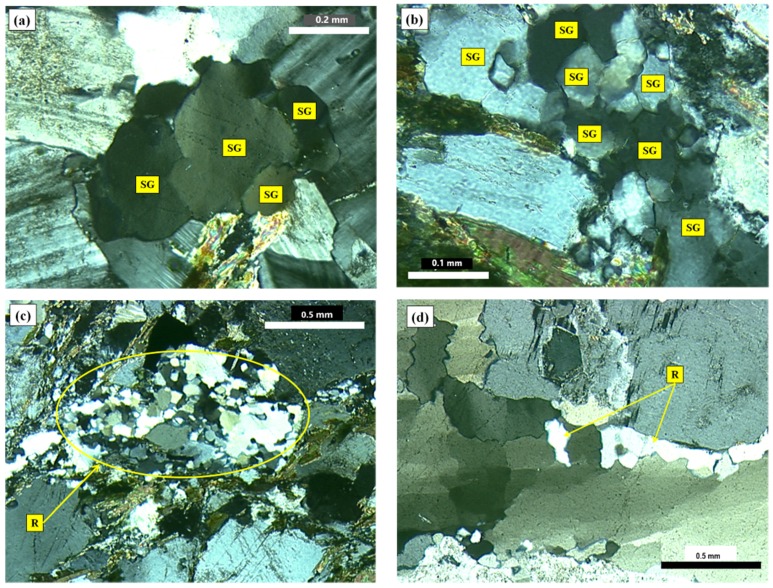
Photomicrographs of thin section under cross-polarized transmitted light microscopy showing the features of degree-3 (**a**,**b**) and degree-4 (**c**,**d**) of quartz deformation: SG = sub-grain, R = recrystallized grain.

The walls that divide the quartz grains into sub-grains are clearly evident at degree-3 of deformation. The sub-grain boundaries can be observed distinctly when optical microscopy is used, as shown in Figure 3a,b. For degrees 1 and 2, it can be verified that the undulatory extinction of quartz is slightly visible but the walls inside the grain are not evident. The recrystallized grains (degree-4 of deformation) behave in a similar way to the innocuous grains, which present no deformation (degree-0 of deformation). Figure 3c shows recrystallized grains from a deformed grain. However, in most samples the recrystallized grains occur only at the edges of the original grains, as Figure 3d shows. Neto et al. [13] also observed a presence of recrystallized sub-grains in deformed quartz.

**Figure 4:**
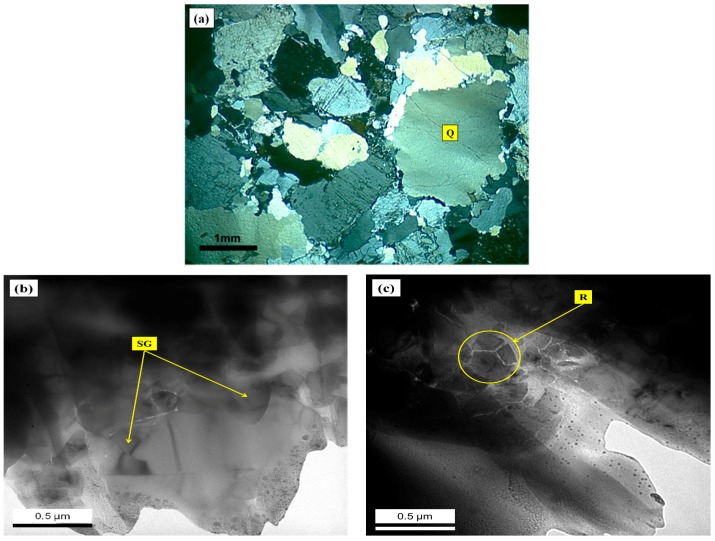
Photomicrographs of thin section of the quartz degree-1 (Q) under cross-polarized transmitted light microscopy (**a**) and transmission electron microscopy (TEM) (**b**,**c**), highlighting the formation of sub-grains (SG) and recrystallized grains (R) of the quartz.

In order to relate the features studied by optical microscopy (Figure 3) to the potential tendency of the samples to expand, the quartz grains featuring degrees 1 and 2 of deformation were analyzed by TEM (Figure 4). The goal was to investigate the presence of deformation walls in these grains, which cannot be visualized when optical microscopy is used. As previously mentioned, the deformation walls tend to weaken the grains, thereby increasing their reactivity.

TEM images of quartz grains featuring degrees 1 and 2 showed nano-walls in regions of quartz where there was a tendency to subdivision. Figure 4 presents micrographic images of a grain with mild undulatory extinction (degree-1). TEM analysis shows the selected grain has displacement walls (Figure 4b,c). According to Putnis [14], displacement is a small flawed line in which the network of atoms breaks within the crystal. The displacement walls are defined by the edge of the flawed network of atoms. The author explains that when the crystal is submitted to stresses, displacements are formed. As a consequence, the networks of atoms move and end up breaking. It is important to point out that when TEM analyses were performed, the breaking of the atomic networks were not frequent in degree-1 of deformation.

However, as Figure 4b,c show, in some areas the formation of sub-grains can be observed. This indicates that even in a mildly deformed grain there are internal paths which are prone to the reaction with the alkaline concrete pore solutions and to the development of ASR. In addition, it is also important to note a feature of the recrystallized quartz grain, as shown in Figure 4c, which presents a very small deformation. The complete recrystallization is attributed to the formation of crystals with perfect hexagonal symmetry. In the hexagonal crystal system, to which quartz belongs, there are three axes of equal length, with angles of 120° [15].

**Figure 5:**
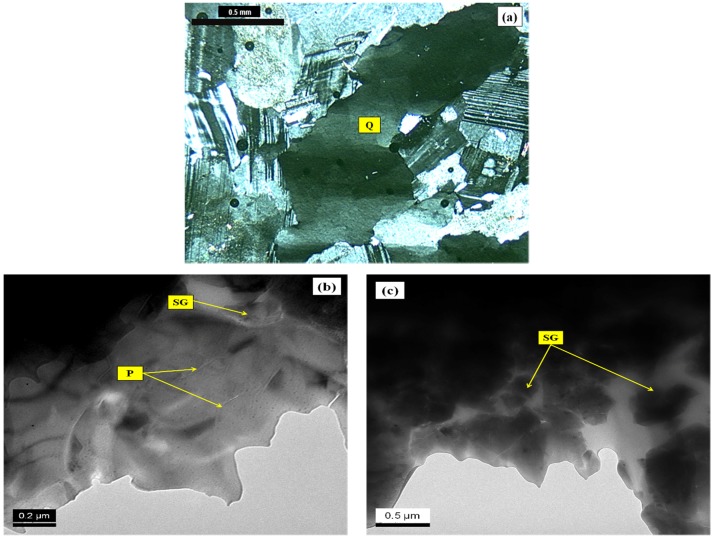
Photomicrographs of thin section of the quartz degree-2 (Q) under cross-polarized transmitted light microscopy (**a**) and TEM (**b**,**c**), highlighting the formation of sub-grains of the quartz (SG), deformation walls (P).

With the growth of deformation, the density of the flawed microstructures enables the linkage of alkali hydroxides and the rupture of the Si-O-Si bonds, thus forming amorphous regions inside the quartz grains. The increase in displacement density was verified in the degree-2 quartz grain (Figure 5b) with TEM analyses. This grain is highly deformed, featuring marked undulatory extinction and deformation bands (Figure 5a). Passchier and Trow [1] explain that the formation of bands occurs when a grain is submitted to stresses, which cause displacements that tend to concentrate in planar zones. As a consequence, the displacement density in other regions of the quartz grain is reduced. In optical microscopy, this can be observed in crystals with distinct areas of extinction. The transition from one area to another is called a deformation band.

The sub-grains shown in Figure 5c were identified in larger proportions in the degree-2 sample due to the increase of the flaws in the crystal structure of this grain. The same occurred with the displacement walls, which were scattered over the analyzed areas (Figure 5b). Wenk et al. [2] compared the displacement density of quartz grains with different degrees of deformation. As in the present research, they deduced that as the deformation of the grains grows, this tends to increase the number of the displacement walls.

Wenk et al. [2] also highlighted the presence of deformation fringes when the deformation of the grain is excessively marked. The presence of displacement fringes hinders the visualization of the displacement walls. The fringes show the intense disorder of the crystal structure. However, the authors noticed that these fringes can often be mistaken for fringes of thickness, which reflect an inadequate thickness of the grain in TEM analysis [26]. In the present study, fringes were observed in some areas but it was not possible to determine if they were a result of the structural disorder or if they were caused by the difference of thickness of the analyzed area.

Therefore, according to the characterization of the degrees of deformation of the samples, any quartz grain has flaws that allow alkaline hydroxides to enter it. As a consequence, any grain has reactive potential and can lead to the ASR developing. Even the slightly deformed grains have displacement walls and also sub-grains, which may not be visible by optical microscopy. The variation between grains featuring distinct degrees of deformation influences the intensity of the occurrence of ASR. The more deformed the grains are, the larger the number of flaws in the structure of the crystal is. After recrystallization, quartz becomes fully recovered and flawless.

The micrographic images published by Mesquita [27] are useful to illustrate how the penetration of alkaline hydroxides occurs in quartz grains and that this leads to the development of the ASR. The author studied the metamorphic-hydrothermal alteration of the quartz in nature and verified that the presence of displacement walls allows fluids to enter that alter the quartz grains. However, in recrystallized grains, fluid inclusions are not observed, that is, in these grains there are no paths along which fluids can penetrate them.

### 3.2. Assessment of Potential Expansion and Quartz Dissolution

In order to relate the potential of quartz grains to dissolve to different degrees of deformation, the visible spectrophotometer test and chemical method of the ASTM C 289/2007 [21] were performed.

**Figure 6:**
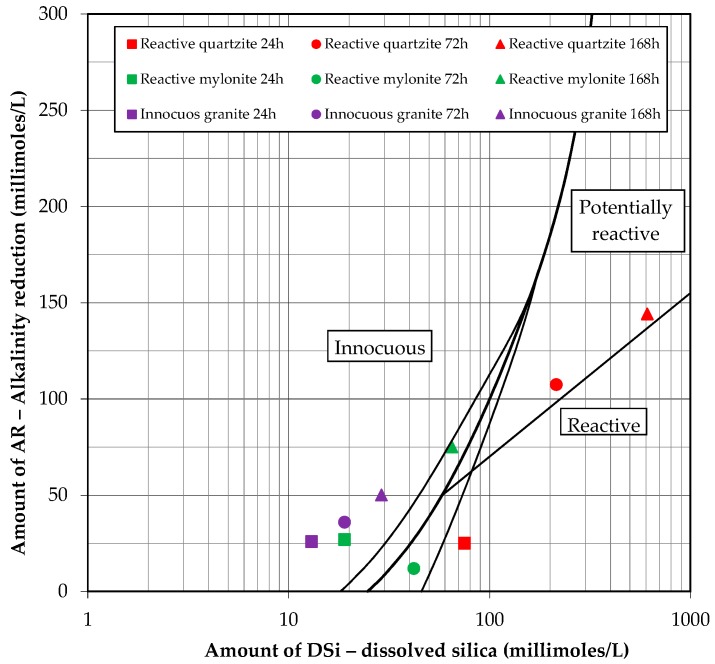
Relationship between the amount of dissolved silica and the reduction in alkalinity of the aggregates submitted to the ASTM C 289/2007 test.

Figure 6 shows the graph that relates the amount of dissolved silica and the reduction in alkalinity for the purposes of classifying the reactive potential of the aggregates under this method [21]. In addition to the analysis of exposing the aggregates to the alkaline solution for 24 h, their behavior was also studied at 72 h and 168 h. Figure 6 shows that the increase in the exposure time of the reactive quartzite and innocuous granite to the alkaline solution resulted, respectively, in increasing the dissolution of silica by 12% and 45%, from 24 h to 168 h and, consequently, in reducing the alkalinity. However, for the reactive mylonite aggregate, there was an increase in the amount of silica dissolved as time passed, but the reduction in alkalinity was lower after 72 h of exposure, and it started to increase again at 168 h.

Thus, what was generally noticed by assessing the reactivity of the aggregate by the chemical method [21] was that the tested variations did not prove effective in better assessing the aggregates, since the quartzite (proven to be reactive in the field) was classified as reactive when exposed for 24 h (the time set by the aforementioned standard) but at 72 h and 168 h it became potentially reactive. Furthermore, the mylonite, which was also responsible for unleashing the ASR in the field, did not prove to be reactive in any of the conditions tested; the innocuous granite, in turn, was the only one that proved to be innocuous, also after the chemical method.

**Figure 7:**
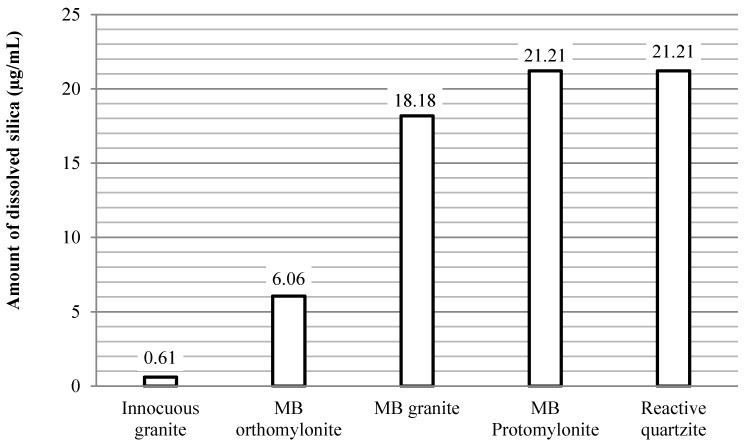
Comparison between the quantities of dissolved silica in the MB rocky outcrop samples, reactive quartzite and innocuous granite.

Therefore, an alternative approach was sought to assess the amount of silica dissolution in the selected samples, using the visible spectrophotometer method [23]. As described in Section 2.2.5, this method was used only with quartz grains extracted from the rocks in order to avoid interference from the other existing minerals. To perform the test an innocuous rock and a reactive rock in the field, as well as expansion tests, were selected (innocuous granite and reactive quartzite). In addition, the quartz grains from the rocks of the Monte Bonito outcrop were also analyzed (MB-orthomylonite, MB-granite and MB-prothomylonite). The results obtained are given in Figure 7.

Figure 7 shows that the quartz from the innocuous granite sample featured a lower dissolution of silica (0.6 µg/mL). This quartz is predominantly deformation-free (degree-0), that is, less prone to dissolution. On the other hand, the silica dissolution measured in the reactive quartzite was identical to that of MB protomylonite (21.2 µg/mL). The characteristic in common of both samples is the predominance of degree-2 of deformation grains, which are very susceptible to reacting with alkali hydroxides. Furthermore, it is also evident that degree-1 grains associated with degree-2 silica dissolution of the MB granite sample have an important role. Thus, the spectrophotometer method seems to be coherent and may be a promising tool to analyze the dissolution of silica in rocks and the possible relationship with reactive potential.

To compare the potential expansion between rocks, the accelerated mortar bar test [19] was performed. Table 5 presents the results of this test at 30 days, corresponding to the average of expansions from three test specimens of each sample and the quantity of quartz identified in the samples.

Table 5 shows that the rocks from the same rocky outcrop (MB granite, MB protomylonite, MB orthomylonite) present similar expansion (~0.16%), as was already expected since the only difference between them is the texture of the grains. Analysis of variance (ANOVA) was used to verify whether these differences can be considered statistically significant throughout the test, that is, to investigate the influence of the age and type of the rocks on the results of the expansion test [19]. According to the ANOVA results, the expansions of the accelerated mortar bar test performed on the rocks from the MB rocky outcrop showed that there were statistical differences between them. In addition, this analysis shows that the type of rock significantly influences the expansions, as do the age and the interaction between type of rock and age, that is, even though they are very similar, the expansions can be considered statistically distinct. This happens because the differences in the expansions in these rocks remain the same from the beginning to the end of the test. Besides, statistical outcomes indicate that, over time, the expansions tend to grow continually.

Regardless of the analysis performed, the slow behavior of the granitic rocks is typical when the silica involved in the reaction comes from the deformed quartz, since this mineral takes longer to react. Besides what was aforementioned and taking into account that even in grains with a small deformation, there are flawed paths which enable the reaction with alkaline hydroxides to occur over time (see Section 3.1), so it can be affirmed that the rocks from the MB rocky outcrop are potentially reactive. It is still important to emphasize that we should not consider only the results achieved by the accelerated mortar bar test to classify the reactive potentiality of an aggregate. Concrete prism tests should be carried out in order to complement this study.

**Figure 8:**
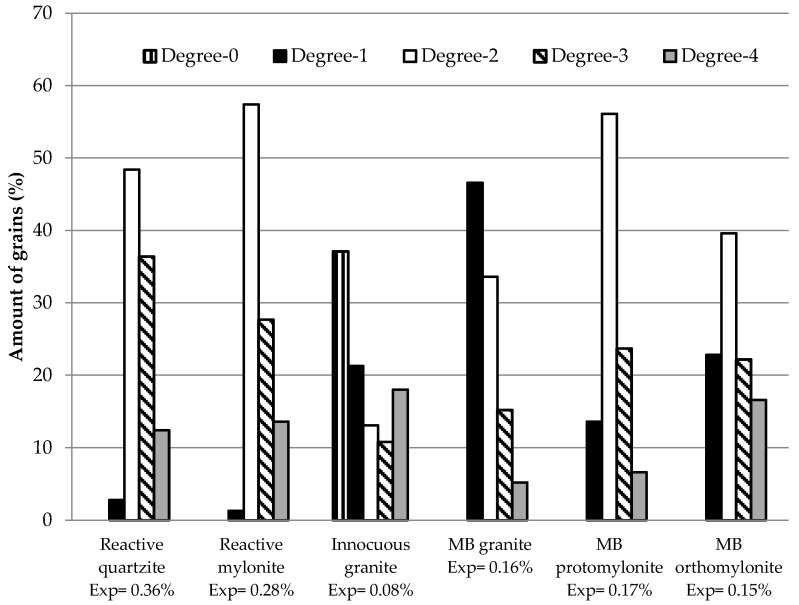
Amount of quartz grains featuring different degrees of deformation in the samples.

As to rocks from the same rocky outcrop, Table 4 shows that the MB orthomylonite presents the highest amount of the sum of degrees 3 and 4 of the quartz grains, which are the grains resulting from the rocks having been deformed more intensely. However, their expansions were lower than those of the other samples, thus showing that the recrystallized grains (degree-4) take longer to react, even with the larger surface available. Their deformations are similar to degree-0 of deformation (quartz is deformation-free). This becomes evident in Figure 8, which compares the amount of quartz grains featuring different degrees of deformation in the samples. Nevertheless, over time and under conditions of high alkalinity (e.g., concrete), even deformation-free grains exhibit higher susceptibility to reacting with alkaline hydroxides [28,29].

Figure 8 shows that the rocks featuring expansions greater than 0.10% in the accelerated mortar bar test (MB granite, MB orthomylonite, MB protomylonite and innocuous granite) present a greater quantity of degree-1 grains than the reactive field rocks classified in this test as potentially reactive (reactive quartzite and reactive mylonite). Degree-1 grains take longer to react with alkaline hydroxides; however, the reaction tends to develop. Knauss and Wolery [29] show that, when pH is higher than 7, quartz loses stability and tends to dissolve over time. In concrete the pH-value is around 12, and this leads to dissolution.

Krauskopf [28] explains that the alkali environment of concrete can destabilize any quartz grain, even those featuring more perfect structures and even though it takes much time. As an example, the study carried out by Fernandes et al. [30] can be mentioned. The authors verified the development of ASR in a Portuguese dam built in 1960, where the quartz in the granite was slightly deformed. Similar results were discussed by Št’astná et al. [31].

The present study shows that all quartz grains interact and contribute to ASR development over time, regardless of their degree of deformation. Thus, even in the rocks in which there undeformed quartz grains (e.g., innocuous granite sample) predominate, the reaction can be developed due to the exposure period of quartz to the high pH of concrete.

### 3.3. Discussion

The ASR has been related to the deformation of grains of quartz occurring in rocks, and is often identified by the undulatory extinction of the grains. However, as some authors have been reporting, the most deformed rocks do not always result in a higher ASR intensity. Monteiro et al. [32] correlated the deformation of metamorphic aggregates to the alkali-silica reaction and verified that in the intensely strained grains, the undulatory extinction may disappear, suggesting that other parameters need to be assessed.

The kinetic of the ASR developed in the structures incorporating the quartzite and mylonite aggregates indicates that the higher the number of degree-2 quartz grains, the faster the occurrence of the ASR in the field; since the structure built with the quartzite was affected by the ASR after 13 years [15] and the structure with mylonite after 12 years [16]. In the accelerated test, it was also verified that the quartzite expanded more rapidly and intensely than the mylonite, the same occurring in the dissolution analyses. However, it is important to be careful when conducting the accelerated test, because of the higher temperature and alkalinity of the test. Thus, it is important to emphasize that there are other facts intervening in favor of the ASR in these structures, such as humidity and the alkali content of the concrete, among others, which are not taken into account in the present study.

It is also relevant to analyze individually the distribution of quartz grains, according to the different degrees of deformation, in the reactive and innocuous rocks in the field. In quartzite, as shown in Table 4, quartz grains with degree 2 of deformation predominate. This rock also has a large number of subgrains (degree 3), which reflects how intensely the rock has been deformed.

Mylonite and Granite were collected from the same region (Recife/PE–Brazil), but they have very distinctive characteristics. Mylonite has a great quantity of degree-2 quartz, that is, it appears markedly strained, and features deformation bands. In the granite rock, on the other hand, there is a prevalence of degree-0, deformation-free grains. Considering the percentage of degree-2 grains quantified for granite, and taking into account that no ASR was observed in the structures in which this rock was used, it can be suggested that it is likely that values lower than 13% of degree-2 suffer minor damage from ASR, that is, apparently, rocks with larger percentage of grains with this petrographic feature are more likely to present ASR. Nevertheless, it would be necessary to collect more field data so as to correlate the specific use of the granite rock and concrete performance related to ASR, according to uncertainness presented in Andrade et al. [16].

Transmission electronic microscopy showed that the quartz grains with deformation bands have internal displacement walls, which could not be detected by conventional petrography using an optical microscope. In future studies it would be useful both to analyze the potential development of the ASR using tests developed with concrete and to compare this with the different degrees of deformation of quartz, and also to study the dissolution of the silica using a visible spectrophotometer and to analyze the results by means of X-ray micro-diffraction, using Synchrotron energy, so as to evaluate the attack of the alkaline hydroxides on quartz grains with different degrees of deformation.

## 4. Conclusions

In the present study a methodology based on petrographic analysis was used to quantify different features of the quartz, which were related to the potential of silica dissolution and development of the expansions resulting from ASR.

The research was verified that rocks comprised mainly of quartz grains featuring deformation bands (degree-2 of deformation) exhibit higher susceptibility to expanding in the accelerated mortar bar test. TEM analysis shows that the quartz grains with deformation bands have internal displacement walls. These are regions in the grain where the chemical bonds of Si and O atoms weaken and form amorphous regions inside the quartz grains, which are more prone to reacting with alkali hydroxides. These walls were also analyzed using TEM on the slightly deformed grains, featuring mild undulatory extinction (degrees-1 of deformation). This indicates that even in a mildly deformed grain there are internal paths which are prone to the reaction with the alkaline concrete pore solutions and to the development of ASR. The more deformed the grains are, the more flaws there are in the structure of the crystal. After recrystallization (degree-4 of deformation), quartz becomes fully recovered and flawless. Those walls could not be detected by conventional petrography using an optical microscope.

Regarding the quartz grains in which the formation of sub-grains was observed, it was verified that they represent an interfacial behavior between the grains with bands and the recrystallized grains, and can significantly contribute to the increase of expansions, especially in the rocks considered less deformed. The recrystallized grains, for their part, are smaller and do not feature deformation, and contribute to the development of the ASR as much as the deformation-free grains.

The silica dissolution of the samples was clearly identified by using the visible spectrophotometer method, thus elucidating that this technique can be successfully used for this kind of assessment. It was also possible to verify that quartz grains featuring deformation bands are also more soluble. The predominant quartz of reactive rocks in the field also has this characteristic, which confirms the great potential of this test for studies of the silica dissolution and its relation with the development of ASR.

## Figures and Tables

**Table 1:** Petrographic characteristics of the samples featuring different degrees of deformation (from petrography analysis).

Rocks	Characteristics
MB Granite	This rock comprises quartz (37.6%), K-feldspar (33.8%), plagioclase (25.2%) and biotite (3.5%). There is a tendency for biotite bands to form. The sizes of quartz grains vary from 1.0 mm to 4.0 mm. Deformation, recovery and recrystallization features are observed mainly in quartz, but also in feldspars and micas.
MB Protomylonite	This rock comprises quartz (28.7%), K-feldspar (39.6%), plagioclase (26.3%) and biotite (5.4%). This rock represents the intermediate degree of deformation. There is a reduction in the size of grains. The average size of the feldspars is 1.5 mm. Quartz shows a thin and lengthy shape, with high undulatory extinction and deformation bands. Small bands of recrystallized quartz are observed.
MB Orthomylonite	This rock comprises quartz (33.3%), K-feldspar (36.5%), plagioclase (24.0%) and biotite (6.3%). There is a reduction in the size of grains, which ranges from 0.5 mm to 1.0 mm. High foliation of feldspars, biotite, micas and quartz bands are observed.

**Table 2:** Rocks selected used in structures with/without alkali-silica reaction (ASR) pathologies.

Rock	Structure	Origin	Characteristics	ASR	
				YES	NO
Quartzite	Furnas Hydroelectric Plant	Goiás/Brazil	This rock comprises quartz (93.5%) and micas (6.5%). The quartz appears highly deformed, with serrated grains, indicating that they have not been recrystallized yet. The size of the quartz grains varies from 0.05 mm to 0.3 mm.	**X**	
Mylonite	Foundations of Residential buildings	Pernambuco/Brazil	This rock comprises quartz (14.9%), K-feldspar (34.8%), plagioclase (12.9%) and micas (38.8%). The grains of quartz vary in size—from 0.01 mm to 1.2 mm, there being a prevalence of smaller grains which are grouped in bands and are highly deformed. They feature sub-grains which in turn are also clustered. The K-feldspars are highly deformed, featuring undulatory extinction and alteration towards micas. Formations of pressure shadows can be seen in the regions where deformations are more intense, and comprise quartz and micas of low crystallinity.	**X**	
Granite	Foundations of residential buildings	Pernambuco/Brazil	This rock comprises quartz (23.9%), K-feldspar (24.2%), plagioclase (36.5%) and biotite (15.4%). The quartz grains are not very deformed; few have undulatory extinction, which is of mild intensity. Their average size is 1.2 mm. The K-feldspars appeared altered to carbonates and micas. There is a tendency for biotite bands to form.		**X**

**Table 3:** Classification proposed to characterize the degrees of deformation of quartz by petrographic analysis.

Deformation Degree	Description	Image
Degree-0 Quartz is deformation-free	Quartz grains free of defects, without deformation. These grains are homogeneously extinguished as the plate of the optical microscope is rotated, indicating that their crystal lattice are free of defects (displacements).	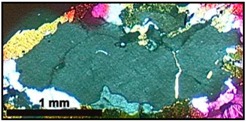
Degree-1 Quartz with mild undulatory extinction	First stage in the deformation process. The quartz grains features undulatory extinction, that is, they are not homogeneously extinguished, and thus retain lighter and darker zones. This feature means that there are some deformations in the crystal lattice.	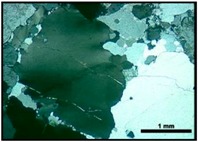
Degree-2 Quartz with marked undulatory extinction, forming deformation bands	Increase in the deformation of quartz. Undulatory extinction is more marked, thus creating well-defined zones inside the crystal (displacement walls start to form). These zones are called deformation bands and are regions where the crystal lattice is deformed, thereby weakening the chemical bonds.	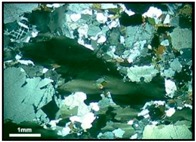
Degree-3 Quartz with marked undulatory extinction, forming sub-grains	Growing grain deformation, widening deformation bands, i.e., an increase in the defects of the crystal lattice, which creates arched regions where chemical links tend to break more easily (sub-grain walls well-defined inside the original grain).	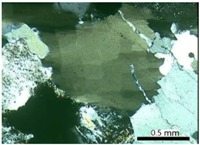
Degree-4 Recrystallized quartz	Last stage of the deformation process. The sub-grains are completely individualized and form new grains, without defects, in a process called recrystallization. The recrystallized grains have the same characteristics observed in the first stage, i.e., they are grains without deformation of the crystal lattice, yet they are smaller in size.	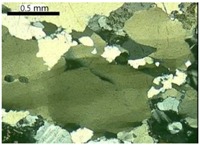

**Table 4:** Quantification of the quartz grains featuring different degrees of deformation (from petrography analysis, counting score of 2000 points per sample—repeated 5 times).

Samples	Number of Grains (%)				
	Degree-0	Degree-1	Degree-2	Degree-3	Degree-4
	Quartz without Deformation	Quartz with Mild Undulatory Extinction	Quartz with Marked Undulatory Extinction, Forming Deformation Bands	Quartz with Marked Undulatory Extinction, Forming Sub-grains	Recrystallized Quartz
MB Granite	-	46.6	33.6	15.2	5.2
MB Protomylonite	-	13.6	56.1	23.7	6.6
MB Orthomylonite	-	22.8	39.6	22.2	16.6
Reactive quartzite	-	2.8	48.4	36.4	12.4
Reactive mylonite	-	1.3	57.4	27.7	13.6
Innocuous granite	37.1	21.3	13.1	10.8	18.0

**Table 5:** Expansion of the accelerated mortar bar test at 30 days.

Sample	30-Day Expansion (%)	ASTM C 1260 Classification	Quartz Quantity (%)
Reactive quartzite	0.36	Potentially reactive	93.5
Reactive mylonite	0.28	Potentially reactive	14.9
Innocuous granite	0.08	Potentially innocuous	23.9
MB granite	0.16	Potentially reactive	37.6
MB protomylonite	0.17	Potentially reactive	28.7
MB orthomylonite	0.15	Potentially reactive	33.3
